# The complete plastome genome of *Incarvillea compacta* (Bignoniaceae), an alpine herb endemic to China

**DOI:** 10.1080/23802359.2019.1681916

**Published:** 2019-10-25

**Authors:** Xiayu Wu, Cuixian Peng, Zhimin Li, Shaotian Chen

**Affiliations:** a School of Life Sciences, Yunnan Normal University, Kunming, China;; b Wenshan Academy of Agricultural Sciences, Wenshan, China;; c College of Pharmaceutical Science, Yunnan University of Chinese Medicine, Kunming, China

**Keywords:** Chloroplast genome, *Incarvillea compacta*, Hengduan Mountain, endemic

## Abstract

*Incarvillea compacta* is a threatened species endemic to the Hengduan Mountain and has been undergoing a successive reduction in the area of occupancy. In the present study, we assembled and characterised the complete chloroplast (cp) genomes of this species. The plastome genome was 150,154 bp in length, and overall GC content was about 40.5%. The circle molecular contained 110 genes including 77 protein-coding genes, 29 tRNA, and four rRNA. Phylogenetic analysis suggested that *I. compacta* is sister to *Tecomaria capensis*.

*Incarvillea compacta* Maxim. is endemic to China and distributed in the Hengduan Mountain (Wang et al. 1990). Owing to the threaten of grazing, this species has been undergoing a successive reduction in the area of occupancy, and has been sorted into VU (vulnerable) category (Chen et al. 2010), based on International Union for Conservation of Nature (IUCN) criteria (version 2.2; Mace and Stuart 1994). In this study, we assembled and characterised the complete chloroplast genome of *I*. *compacta*, which is the firstly released cp genome of the genus *Incarvillea* Juss.

The plant material was obtained from Bangda, Changdu County, Tibet (97°4′32.38″E, 30°35′50.21″N), and the voucher (C11617) was deposited in the Museum of Ethnic Medicine, Yunnan University of Chinese Medicine. We extracted the total DNA using a modified CTAB method (Doyle and Doyle 1987), and then amplified the chloroplast genome using nine universal primer pairs following the recommended protocol (Yang et al. 2014). The amplification product was sequenced using the Illumina Hiseq 2000. Finally, we aligned, assembled, and annotated the plastome of the species using Geneious 8.1 (Kearse et al. 2012), referring to the plastome of Tecomaria capensis (GenBank Accession no.: MG831880.1). The length is 150,154 base pairs (bp) for the complete chloroplast genome of *I. compacta*, and the overall GC content was about 40.5%. The plastome exhibited a typical quadripartite structure, an LSC (large single copy) region of 81,527 bp and an SSC (short single copy) of 21,925 bp, linked by two inverted regions (IR) of 23,351 bp. The GC content values of LSC, SSC, and IR regions are 39.1%, 35.9%, and 45%, respectively. The whole chloroplast genome was composed of 110 genes, including 77 protein-coding genes, 29 tRNA genes, and four rRNA genes. We have submitted the annotated complete chloroplast genome to GenBank and the accession number was MN446000.

To further infer the phylogenetic position of *I. compacta*, other 12 complete chloroplast genome sequences were selected for phylogenetic analysis, including *Neojobertia candolleana* (MG008316), *Podranea ricasoliana* (MG831877), *Adenocalymma allamandiflorum* (NC036494), *Amphilophium chocoense* (MK415793), *Crescentia cujete* (KT182634), *Dolichandra cynanchoides* (MG831874), *Pyrostegia venusta* (MG831878), *Sampaiella trichoclada* (MG831879), *Tanaecium tetragonolobum* (KR534325), *Tecomaria capensis* (MG831880), *Anemopaegma arvense* (MG831872). We aligned all 13 chloroplast genome sequences using Geneious 8.1 (Kearse et al. 2012), and a neighbour-joining tree (Saitou and Nei 1987) was constructed using MEGA7 (Kumar et al. 2016) with 1000 bootstrap replicates. The result shows that *I. compacta* is sister to *Tecomaria capensis* (Figure 1), which indicated the relationship between two genera, *Incarvillea* and *Tecomaria*, is not consistent with other study (Olmstead et al. 2009).

**Figure 1.**
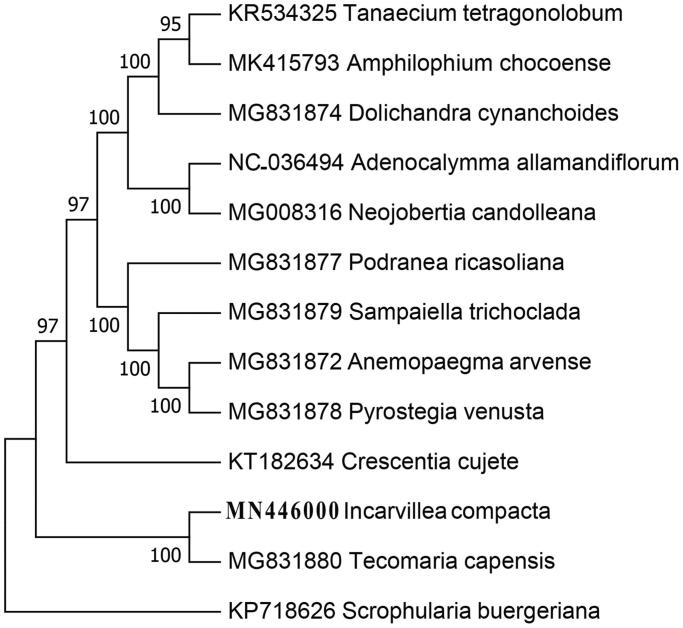
Phylogenetic position of *Incarvellea compacta* inferred from ten chloroplast genomes. Bootstrap support is indicated for each node.

Our studies presented the first complete chloroplast genome of the herb genus *Incarvillea*, and it will facilitate the further investigation on the genus based on chloroplast DNA data.
